# The order of sequential exposure of U2OS cells to gamma and alpha radiation influences the formation and decay dynamics of NBS1 foci

**DOI:** 10.1371/journal.pone.0286902

**Published:** 2023-06-12

**Authors:** Adrianna Tartas, Lovisa Lundholm, Harry Scherthan, Andrzej Wojcik, Beata Brzozowska

**Affiliations:** 1 Biomedical Physics Division, Faculty of Physics, University of Warsaw, Warsaw, Poland; 2 Department of Molecular Biosciences, The Wenner-Gren Institute, Stockholm University, Stockholm, Sweden; 3 Bundeswehr Institute of Radiobiology Affiliated to the Univ. of Ulm, Munich, Germany; 4 Institute of Biology, Jan Kochanowski University, Kielce, Poland; Universidad de la Republica Uruguay, URUGUAY

## Abstract

DNA double strand breaks (DSBs) are a deleterious form of DNA damage. Densely ionising alpha radiation predominantly induces complex DSBs and sparsely ionising gamma radiation—simple DSBs. We have shown that alphas and gammas, when applied simultaneously, interact in producing a higher DNA damage response (DDR) than predicted by additivity. The mechanisms of the interaction remain obscure. The present study aimed at testing whether the sequence of exposure to alphas and gammas has an impact on the DDR, visualised by live NBS1-GFP (green fluorescent protein) focus dynamics in U2OS cells. Focus formation, decay, intensity and mobility were analysed up to 5 h post exposure. Focus frequencies directly after sequential alpha → gamma and gamma → alpha exposure were similar to gamma alone, but gamma → alpha foci quickly declined below the expected values. Focus intensities and areas following alpha alone and alpha → gamma were larger than after gamma alone and gamma → alpha. Focus movement was most strongly attenuated by alpha → gamma. Overall, sequential alpha → gamma exposure induced the strongest change in characteristics and dynamics of NBS1-GFP foci. Possible explanation is that activation of the DDR is stronger when alpha-induced DNA damage precedes gamma-induced DNA damage.

## Introduction

The reaction of cells to DNA damage, termed DNA damage response (DDR), is a complex multistep process. The structure and spatial distribution of DNA lesions, especially DNA double strand breaks (DSBs), which are a particularly deleterious form of DNA damage [1], dictate the selection of pathways which form the DDR [2–4]. DSBs induced by ionizing radiation activate the MRE11-RAD50-NBS1 (MRN) complex, where the role of the Nijmegen Breakage Syndrome 1 (NBS1) protein is to sense the DSBs, to activate ATM and to ensure stability of the complex at the DNA damage site to facilitate DNA repair and pathway choice [3, 5–8]. Upon binding to a DSB, ATM, DNA-PKcs or ATR phosphorylates the histone H2AX at serine 139 of its C-terminus (termed γ-H2AX) [9], a step that leads to the recruitment of the adaptor/mediator p53-binding protein 1 (53BP1) or BRCA1, depending on the choice of the repair pathway, that is required for processing the DDR signal and to act as a platform for the recruitment of other repair factors [10, 11]. Activated DDR proteins accumulate at damaged sites forming microscopically visible subnuclear foci [12]. The frequency of foci is proportional to the level of damage [13], but it is also influenced by factors that modify DDR signalling [14–16].

Among various DNA damaging agents used to study the DDR, ionising radiation takes a specific position because of its ability to directly induce DSBs. Based on ionisation density along its track, radiation is classified as sparsely or densely ionising [17]. Examples of the former are photons and electrons, while examples of the latter are alpha particles and heavy ions. The ionisation density is defined by the linear energy transfer (LET) that describes the mean amount of energy lost by an ionisation particle per unit distance as it traverses matter [18]. Per unit dose, high and low LET radiation induces the same number of ionisations inside a cell. However, because the ionisation events are concentrated along a particle trajectory, the same dose of high LET radiation is delivered in few tracks as compared to dispersed ionization events in the case of low LET radiation [19]. The dense ionisation of high LET radiation results in a higher DSB density and complexity in a unit volume as compared to low LET radiation [19]. The same DNA repair pathways are activated in cells exposed to high and low LET radiation [20, 21], but their proportions may diverge. Depending on the position of the cell in the cell cycle, DSB complexity as well as the local chromatin landscape, there are canonical or alternative non-homologous end joining (NHEJ) and homology-directed repair (HDR) [22]. Complex DSBs pose a particular problem for the DNA repair machinery and their repair is slower and more error prone than the repair of simple DSBs [1, 22–26]. Importantly, high LET radiation induces damage in the DNA mainly by direct ionisation of the molecule. In contrast, the majority of low LET-induced DNA lesions arise indirectly, via radicals predominantly resulting from the radiolysis of water [27]. Tightly packed chromatin protects the DNA against low LET radiation by reducing access for radicals, while sensitising against high LET radiation by constraining DNA repair [28]. Loosely packed chromatin has a reverse impact [29]. Changes in chromatin packing in response to DSBs and its remodelling facilitates access of the DNA repair machinery to DNA lesions [30]. Induction of DSBs leads to altered chromatin dynamics and chromatin motion that may impact the outcome of the DDR [31]. Monitoring of chromatin motion is possible using time-lapse florescence microscopy with sufficient spatial resolution [32].

An interesting question for understanding DDR mechanisms is how the cellular DNA repair machinery copes with damage induced by combined exposure to densely and sparsely ionising radiation. Is the effect simply the sum of effects from the single radiations or does the damage induced by one type of radiation sensitise the cells to the action of the other type? An interaction between both types of radiation could result from DDR engagement in the repair of high LET-induced complex lesions to such an extent that low LET damage is not properly repaired. In support of this, it has been observed that high LET alpha particles induce numerous DSBs along the particle tracks [24, 33, 34] that may sequester limiting factors of the DDR, impeding repair of subsequent damage in such cells [35] and may direct the DNA repair pathways to resection-dependent NHEJ or HDR sub-pathways [36]. Alternatively/additionally, it may be envisaged that dispersed simple DSB lesions could be repaired first thereby influencing the repair of complex damage. Furthermore, it also seems possible that the high LET damage leads to an altered chromatin conformation, which may make the DNA more susceptible to radicals induced by low LET radiation.

In order to study the possible interaction of high and low LET radiation on cells, we have built a dedicated exposure facility where cells in culture can be simultaneously exposed to alpha particles and X-rays [37, 38]. The results of experiments with different cell types and different endpoints quite consistently show that the effect of combined exposure to alpha particles and X-rays is stronger than expected based on simple additivity [37–44]. The experimental results were confirmed by Monte Carlo simulations performed with the PARTRAC tool [45]. However, the mechanisms responsible for the interaction remain largely enigmatic.

If high LET radiation induces an opening of chromatin making the DNA more susceptible to radicals generated by the low LET radiation or the DDR engages in the repair of high LET-induced complex lesions to such an extent that low LET damage is not properly repaired, then a stronger effect would be expected in cells exposed to alpha radiation followed by photon radiation, than following the reversed scenario. The present investigation was designed to test this hypothesis. To this end we exposed U2OS cells to similar doses of alpha and gamma radiation alone or to both radiations in combinations of alternating sequence. It was particularly interesting to study the DDR at high resolution by following the behaviour of individual DNA repair foci. This was possibly by live cell imaging using U2OS cells that stably express the NBS1 protein that is tagged with GFP—green fluorescent protein [46]. NBS1 is a component of the MRN complex that reacts very early to DSB induction, orchestrating the DDR. Thus, the dynamics of NBS1 foci formation can be regarded as a marker of the DDR [7, 8].

DSB-indicating NBS1 foci induced by alpha radiation, gamma radiation and a sequential combination of both (in alternating orders) were followed by live imaging for 300 min. We analysed focus numbers, their areas, intensity and mobility. This time frame was chosen as it allows to monitor the initial 5 h of DSB repair while allowing for recording at a sufficiently high frame rate before the illumination light becomes toxic for the cells. The study extends our earlier investigation where we analysed the live formation and decay of 53BP1 foci in U2OS cells simultaneously exposed to alpha and photon radiation up to 80 min [47]. Beside the analysis of NBS1 and not 53BP1, the major difference between the earlier and present experiment is that the cells were exposed sequentially and not simultaneously to alpha particles and photons, and were followed for 300 min.

## Materials and methods

### Cell culture

All experiments were carried out with human bone osteosarcoma epithelial cells (U2OS) that stably express NBS1-GFP. The cells were previously described and characterized by Lukas et al. (2003) and were kindly provided by Dr. Claudia Lukas from the Novo Nordisk Foundation Centre for Protein Research, University of Copenhagen, Denmark. Cells were cultured in a humidified incubator at 37°C with 5% CO_2_. Cells were grown in Dulbecco’s Modified Eagle Medium (Sigma-Aldrich, D6046, Stockholm, Sweden) supplemented with 10% bovine calf serum (Thermo Scientific, cat. no SH30072.3, Stockholm, Sweden) and 1% penicillin-streptomycin. Two days before irradiation, 10^5^ cells were seeded on 25 mm diameter round glass coverslips.

### Irradiation

Irradiation was performed using an alpha and a gamma radiation source, both available at the Department of Molecular Biosciences, The Wenner-Gren Institute, Stockholm University. Exponentially growing cells were irradiated with 1.7 Gy of alpha particles, 2 Gy of gamma radiation and a combination of 0.83 Gy of alpha and 1.02 Gy of gamma radiation (corresponding to 45% of alpha and 55% of gamma radiation of the total dose of 1.85 Gy) applied in alternating order. The doses were selected to give a clear cellular response. The reason for the imbalance between the alpha and gamma doses results from an error in calculating the alpha dose rate that was discovered after the experiments were finalised. The sequential exposure to both radiations was performed in two ways. In the first scenario, alpha particles were applied first, and then, after ca 5 min needed for transferring cell dishes from one source to the other, the cells were irradiated with gamma radiation. In the second scenario, the reverse order was used. Gamma radiation was delivered with a Scanditronix IC900 irradiator (Scanditronix AB, Uppsala, Sweden), containing two opposing 16.65 TBq ^137^Cs sources that emit gamma rays with a peak energy of 662 keV, yielding a uniform radiation field in the cell exposure chamber. The source was calibrated using Fricke dosimetry in collaboration with the Swedish National Metrology Laboratory at the Swedish Radiation Safety Authority and was 0.372 Gy/min at the time of the experiments. The AIF 08 alpha source with ^241^Am isotope as an alpha emitter (AP1 s/n 101; Eckert and Ziegler, Berlin, Germany) has an activity of (50.0 ± 7.5) MBq and provides alpha particles at a fluence of 23,800 ± 4,600 particles per second and cm^2^ without collimation. The resulting dose rate was 0.223 Gy/min.

For gamma irradiation, the coverslip with cells was coated with 250 μl of cell culture medium, covered with 2.5 μm Mylar foil and placed inside the cell exposure chamber. Irradiation time was controlled according to the instructions of the manufacturer. For alpha irradiation, the coverslip with cells and Mylar foil was placed on a motor-driven shelf that moved the cells towards the source. The irradiation started at the moment when the cover slip touched the source and was stopped by lowering the shelf. Both irradiations took place at room temperature.

The irradiation time needed to deliver 1.7 Gy of alpha radiation was 7 min 32 s. It took 5 min 37 s to deliver 2 Gy of gamma radiation. The pure exposure time to 1.85 Gy of mixed beams was 6 min 33 s (corresponding to half of the summed radiation times for alphas and gammas alone) but it took ca 5 min to transfer a cell sample from one source to another. Hence, the total exposure time to mixed beams was ca 10 min 33 s (approximated to 11 min).

### Live cell imaging

Immediately after irradiation, coverslips with irradiated cells were placed in a pre-warmed (37°C) stainless steel live cell chamber as described in [47, 48]. The chamber, the size of a standard microscopic slide, had a bottomless well in which the round coverslip was positioned and sealed by a Sykes-Moore gasket (O-ring and a plastic nut). This well was filled with pre-warmed growth medium supplemented with sodium pyruvate and 10 mM HEPES (Applied Chem GmbH, A3268, Hamburg, Germany) for pH stability. A coverslip was loosely placed on top of the filled well to prevent evaporation of the medium. Care was taken to avoid air bubbles. The live cell chamber was assembled immediately after irradiation, a process which took ca 4 min and time-lapse microscopy started thereafter. It was performed at 37°C inside a self-constructed temperature-controlled microscope environmental chamber mounted on a Carl Zeiss Axiovert 200 inverted microscope (Jena, Germany). Cell nuclei observed under the microscope were randomly selected and NBS1-GFP fluorescence was excited by 120 ms exposure to 488 nm light from a Polychrome IV monochromator (TILL Photonics, Gräfelfing, Germany) and registered by a CCD camera (PCO Sensicam, Kelheim, Germany). Images were recorded at 1-minute intervals over 300 min, with 120-ms-per-frame exposures. On average, two nuclei were followed per recording session. The images were recorded using the TILL Photonics Imaging System software (TILLvisION v3.3, TILL, Gräfelfing, Germany) installed on a connected PC.

### Image analysis and presentation of results

Images were analysed using the Imaris Microscopy Image Analysis Software (versions 9.9.0 and 9.1.2, Bitplane AG, Oxford Instruments plc). Each cell nucleus was analysed separately. An area in which a nucleus was located was isolated, and its centre of mass position was calculated at a given point in time. The average number of repair foci per nucleus was determined as the sum of all registered foci divided by the number of all imaged nuclei for a given treatment and time of analysis. For graphs showing the mean area of the repair foci for which the data is represented in relative terms, the area was defined as the ratio of a given focal area value to the maximum area recorded. Normal distribution curves with characteristic parameters (mean value and standard deviation) were fitted to the experimental data. 8^th^ order polynomials were fitted to the data representing the change in the area of the foci as a function of time. Uncertainties are from determining the curve fitting parameters. The average relative intensity of the repair foci was determined using the following formula:

$$I=\frac{I_{\text{focus}\;}}{I_{\text{nucleus}\;}-I_{\text{focus}\;}}$$ (1)

where *I*_*focus*_ is the total intensity over the focus area and *I*_*nucleus*_ is the intensity over the entire area of the cell nucleus.

The change in the position of a repair focus was determined by tracking its coordinates and determining the mean square displacement (MSD) [49] which is the square of the path length. The MSD was calculated for the position in the first minute of observing the cell nucleus *d* (*t*_0_) with respect to a reference position over time *d* (*t*_0_ + Δ*t*):

$$MSD={\left(\left[d\left(t_{0}\;+\;\Delta t\right)\;-\;d\left(t_{0}\right)\right]\right)}^{2}.$$ (2)

The function of the subdiffusion model was fitted to the data, for which parameters diffusion radius (*r*_*c*_) and a diffusion coefficient (*D*_*c*_) were determined. *D*_*c*_ is a measure of focus mobility, *r*_*c*_ is a radius within which this focus moves, and d is the dimension of the analysed space (equal 2). This model is given by the following formula:

$$MSD(\Delta t)\;=\;r_{c}^{2}\left\{1-exp\;\left(-\frac{2dD_{c}\Delta t}{r_{c}^{2}}\right)\right\}.$$ (3)

Approximately 30 movies were recorded for each treatment, and the average number of nuclei in one movie is two. A different number of cell nuclei was qualified for the analysis depending on the treatment used. 18 cell nuclei irradiated with alpha particles, 28 with gamma radiation, 25 successive mixed beams with alpha particles first, and 16 successive mixed beams with gamma radiation first were analysed. In the case of the control cells, 22 nuclei were investigated.

Gamma and alpha radiation alone is denoted as γ and α, respectively. Sequential exposure to both radiation types is denoted as γ → α or α → γ reflecting the order to irradiation.

### Statistical analysis

All statistical analyses of the data obtained from the analysis of images was done in Python 3 software (version Python 3.7.9, Python Software Foundation) using additional libraries such as SciPy and Matplotlib. For statistical evaluation, the data were compared using the ANOVA test using Tukey’s post hoc test (focus area and intensity) and the t-test for the means of two independent samples of scores (focus area in two time intervals). The p-values less than 0.05 were considered statistically significant. Data are represented as means ± standard error of the mean (SEM).

In the case of comparing two slope coefficients described by a linear function (focus frequency and intensity), resulting from fitting the following equation: *y* = *at* + *b*, the t-test was used.

## Results

We studied the response of U2OS cells to γ and α as well as a combination of both radiation types by analysing, by live cell microscopy, the formation and disappearance of DSB-indicating NBS1-GFP foci. NBS1 has been shown to associate with localised chromatin domains at DSBs where it is retained [46], allowing DSB damage regions to be followed as NBS1-GFP foci over time. Since little is known about the impact of exposures to mixed IR fields or the impact of the order of successive exposures with different LET with regard to the DDR, we recorded time-lapse movies of U2OS cells exposed to alpha and gamma radiation alone, and a sequential combination of both radiations in alternating order. Image analysis was applied to obtain quantitative and qualitative parameters for NBS1 foci. Due to time needed for placing cell samples in the live cell imaging chamber, recording of images started about 4 min after terminating the irradiation and lasted for a period of 300 min.

### Kinetics of focus formation and decay

**Fig 1:**
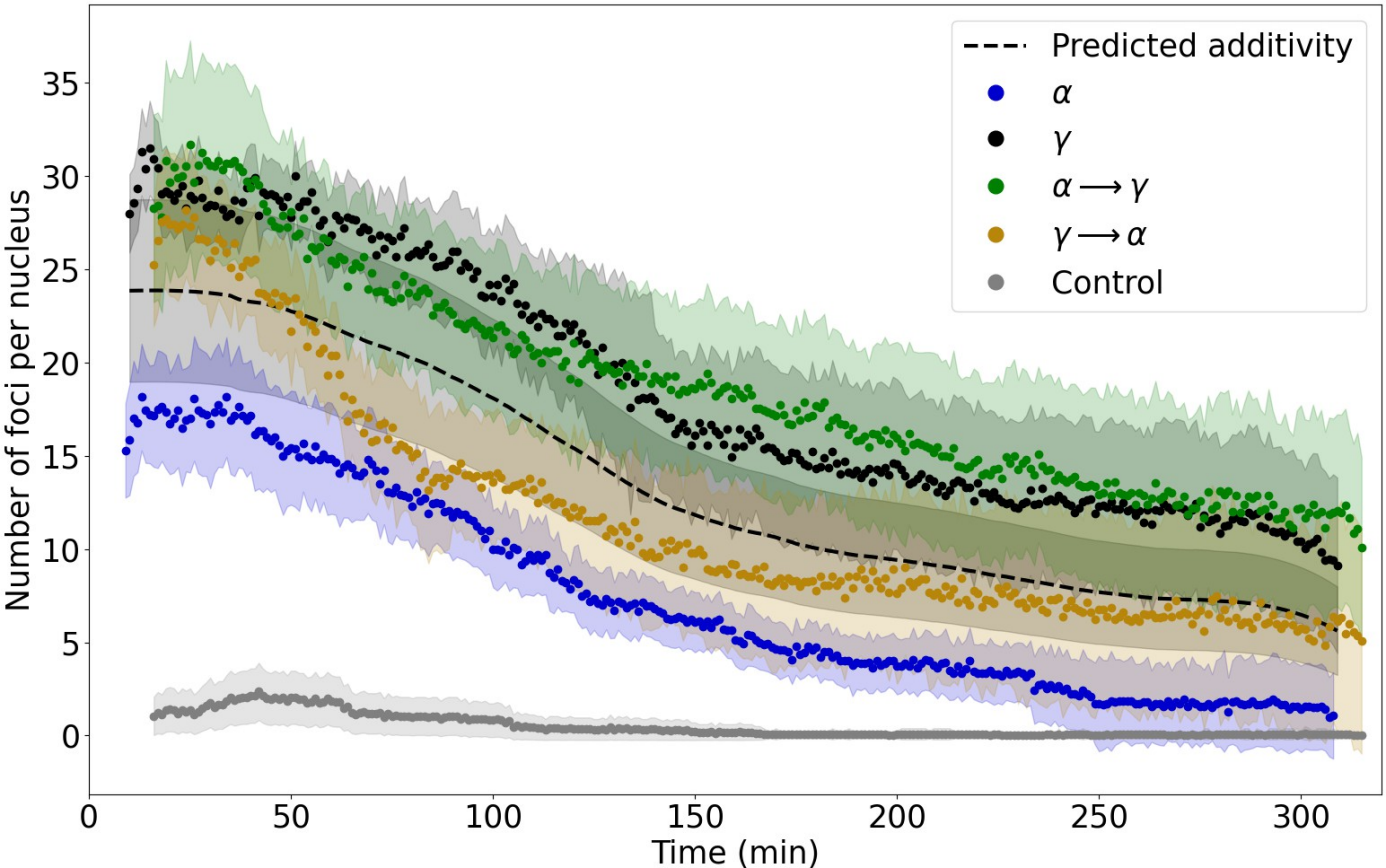
Kinetics of NBS1 focus frequency in control and irradiated cells. Dots represent the experimental data points and the shaded corridors represent the standard errors of the mean (SEM). Time 0 denotes the start of irradiation. Images were acquired every minute for a period of 300 min. The expected frequencies calculated on the basis of the additivity assumption are displayed as a black dashed line. A statistically significant difference (one-way ANOVA) was obtained for all pairs of treatments, except for foci formed after irradiation with γ versus α → γ.

The kinetics of the formation of NBS1-GFP repair foci per cell and their decay is presented in Fig 1 and quantitative parameters are listed in Table 1. A factor that must be considered when projecting all results on a single X-axis corresponding to time of DNA damage repair is that focus formation may start already during radiation exposure and before the start of image acquisition. The exposure time (in min:s) to γ and α alone and the combination of both was 7:32, 5:37 and 10:33, respectively. The time needed to prepare the irradiated cells for image acquisition was 4 min. Hence, in order to meaningfully compare the focus kinetics, time 0 denotes the start of an irradiation. The time of the last acquired image corresponds to 300 min plus the irradiation time and 4 min of preparation time for imaging.

**Table 1:** Observed frequencies of NBS1 foci per cell nucleus in control cells, cells exposed to α and γ radiation alone, to α → γ and γ → α, as well as predicted values based on assumption of additivity. The initial number of foci per nucleus is reported as the mean number of foci recorded in the 16^th^ minute after start of irradiation (16^th^ minute is the first minute of image series recorded for combined irradiations). The final number of foci per nucleus denotes the mean number of foci recorded in the 310^th^ minute, which is the last minute of image acquisition of cells treatment with γ.

Treatment	Mean RIF per nucleus at start of recording	Highest mean number of RIF per nucleus (@ time point)	Mean RIF per nucleus at end of observation
Control	1.1 ± 2.1	2.4 ± 3.1 (36 min)	0.0
α	17.1 ± 2.7	18.1 ± 2.9 (13 min)	1.1 ± 2.3
γ	30.9 ± 2.3	31.5 ± 2.5 (14 min)	9.1 ± 4.7
α → γ	28.3 ± 5.0	31.7 ± 5.6 (25 min)	11.9 ± 5.1
γ → α	25.2 ± 3.3	28.2 ± 3.5 (24 min)	6.4 ± 5.7
Predicted α + γ	23.9 ± 4.8	23.9 ± 4.8 (20 min)	6.5 ± 2.6

The average background number of foci per non-irradiated cell was 1.0 ± 1.8. Irradiation with γ, α and mixed beams induced a significantly elevated number of NBS1-GFP foci (referred to as radiation-induced foci—RIF) per cell as compared to the control (Table 1). RIF frequencies decreased with repair time. The highest average number of RIF was observed in cells exposed to γ. It peaked at 14 min after time 0, with 31.7 ± 5.6 foci per cell. The generally lowest level of RIF was observed following α, with the maximum number of 18.1 (± 2.9) at 13 min post time 0. For mixed beams, the highest mean number of RIF was generally close to the value for γ alone (Table 1). For α → γ the peak RIF value of 31.7 ± 5.6 was observed at 25^th^ min. For γ → α the time to reach the maximum number of repair foci was similar (24 min), but the observed value of 28.2 ± 3.5 RIF was slightly but insignificantly smaller.

In the case of mixed irradiation, there was a noticeable difference in the kinetics of RIF between α → γ and γ → α. After achieving relatively similar numbers at around 25 min post time 0, the numbers decreased over time at different rates. For γ → α a more rapid decline of RIF was observed as compared to α → γ (Fig 1). The slope of the linear function describing the first 90 min of RIF decay after γ → α was approximately two times steeper than for α → γ (see Table A1 in S1 Appendix for the fit parameters). For comparison with achieved results, a theoretical curve was drawn to describe the expected RIF values resulting from an additive action of 0.83 Gy α plus 1.02 Gy γ. During the first 50 min post time 0, the number of RIF for both α → γ and γ → α was higher than the predicted number (Fig 1), indicating synergy in the induction of DNA damage. After 50 min, the number of α → γ RIF remained above the expected values, even surpassing the γ level after 140 min. The γ → α RIF numbers fell below the additivity curve 50 min and remained lower until 300 min post time 0.

### Kinetics of focus area

**Fig 2:**
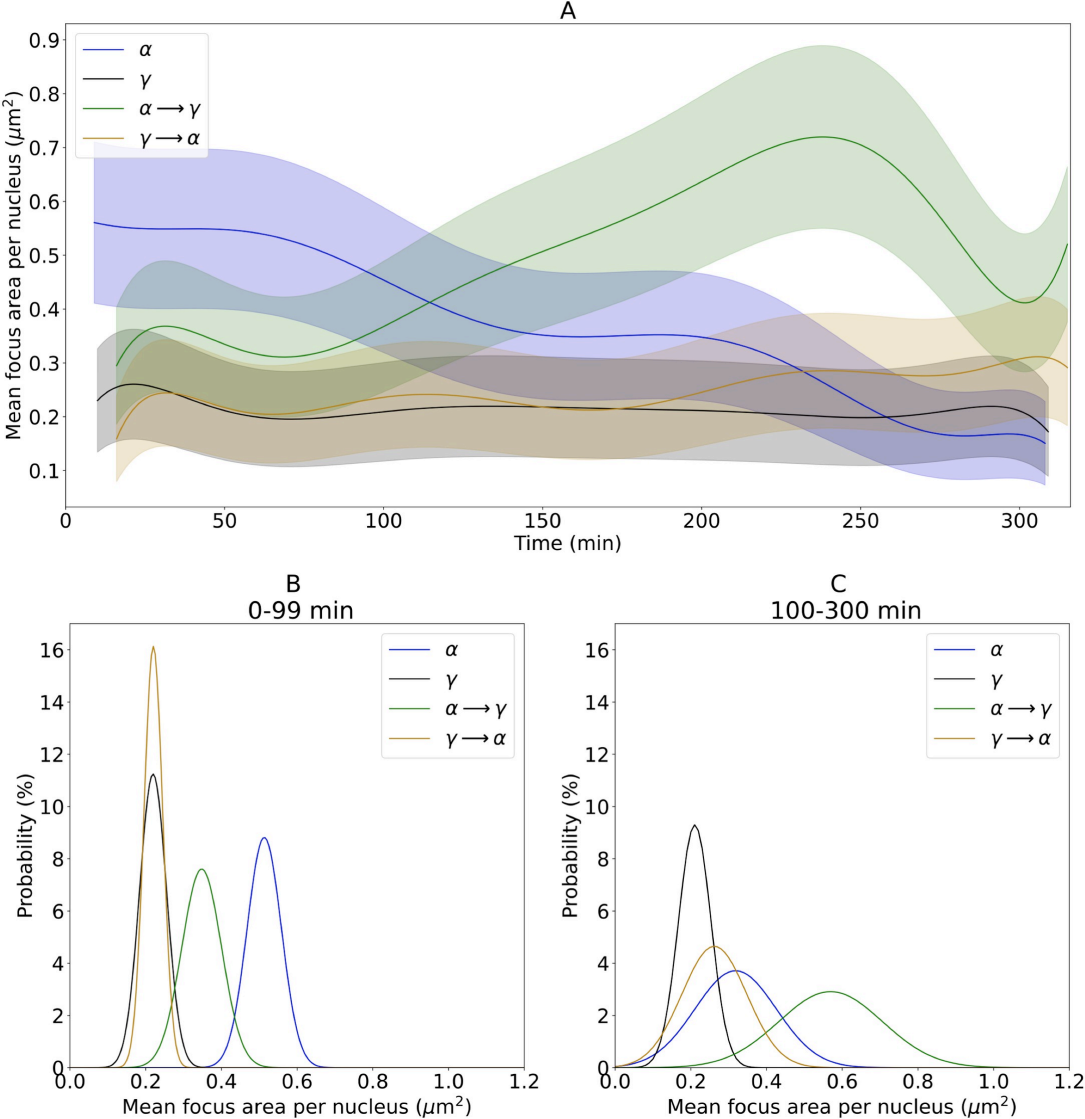
Kinetics of radiation-induced focus area described by polynomial fits (panel A). Corridors represent the uncertainties of fits. Data points are omitted for the sake of clarity but complete graphs can be found in supporting information material (Fig B1 in S2 Appendix). Area distributions of foci detected during 0–99 min are shown in panel B and during 100–300 min in panel C.

In addition to focus numbers, we followed changes of RIF areas. The rational for this analysis is the assumption that the RIF size is directly proportional to DNA damage complexity, being larger after α as compared to γ . Fits of eighth-order polynomial function for the change in the mean RIF area over time are presented in Fig 2 (see S2 Appendix for fit parameters and the experimental data). The largest initial RIF were induced by α, and their area decreased as a function of time. γ and γ → α induced the smallest RIF whose area did not change significantly during the first 180 min of imaging. After this time, there was a slight increase in the area of γ → α RIF, an effect that can be attributed to the remaining of few RIF with complex DNA damage at that time point. Nevertheless, their area was distinctly smaller than that of RIF induced by α → γ. The α → γ induced RIF were initially smaller than those that appeared after exposure to α only. From 150 min onward the α → γ RIF area began to increase from about 60 min to reach a maximum at 270 min post time 0 and was largest among all treatments.

The distributions of RIF areas observed during 0–99 and 100–300 min post time 0 are presented in Fig 2B and 2C and the parameters for fitting the normal distribution function are listed in Table 2. During the first 99 min, RIF induced by γ and γ → α had the same smallest mean area of approximately 0.2 μm^2^. The largest RIF were induced by α (0.513 ± 0.045 μm^2^), while α → γ induced RIF of intermediate size (0.348 ± 0.052 μm^2^). After about 90 min, the mean area of α RIF began to decrease, while the area of γ RIF remained unchanged, being the smallest during most of the observation time. Interestingly, the area of RIF induced by both γ → α and α → γ increased with time, indicating accumulation of complex DNA damage with open DSBs in these foci. For both α → γ and γ → α, the mean RIF area was significantly larger during 100–300 as compared to 0–99 min post time 0 (0.348 ± 0.052 μm^2^ versus 0.60 ± 0.14 μm^2^ and 0.220 ± 0.025 μm^2^ versus 0.262 ± 0.086 μm^2^, respectively).

**Table 2:** Fit parameters of normal distributions describing the focus area histograms. Uncertainties indicate standard deviations. Differences between the early and late interval are considered statistically significant at p <0.05.

Treatment	Area in the time interval 0–99 min (μm^2^)	Area in the time interval 100–300 min (μm^2^)	p-value
α	0.513 ± 0.045	0.32 ± 0.11	< 0.0001
γ	0.219 ± 0.035	0.210 ± 0.043	0.07
α → γ	0.348 ± 0.052	0.60 ± 0.14	< 0.0001
γ → α	0.220 ± 0.025	0.262 ± 0.086	< 0.0001

### Dynamics of focus intensity

**Fig 3:**
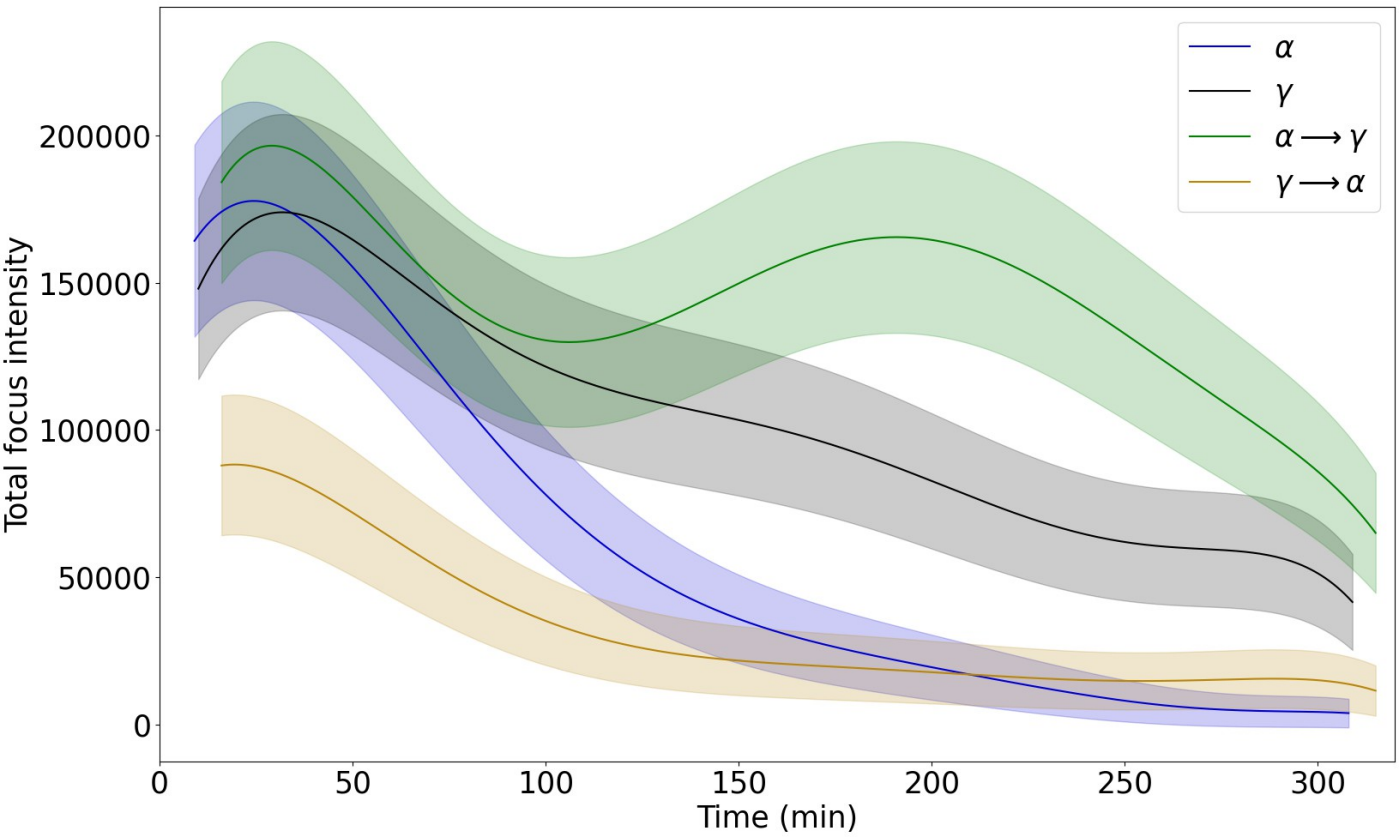
Polynomial functions fitted to average total focus intensities per cell nucleus during 300 min post time 0. Corridors represent uncertainties of fit. A graph including the data points can be found in the supporting information (Fig C1 in S3 Appendix).

Next, we determined the average cumulative intensity of focal NBS1-GFP fluorescence per whole-nucleus as an estimate of the total load of repair proteins (referred to as total RIF intensity). The fit of sixth-order polynomial function for the change in the total RIF intensity over time is presented in Fig 3 (see S3 Appendix for the fit parameters and the experimental data). The shape of the function is similar to that shown in Fig 2A because focus intensity correlates with the number of pixels and thus focus area. The highest values of total RIF intensity were observed for α → γ and, similarly to focus area, a second peak was seen at around 200 min indicating reassortment of the NBS1 protein to large foci at this late time point. The smallest values were seen for γ → α. The strongest decrease in the total RIF intensity over time is observed for cells irradiated with α.

**Fig 4:**
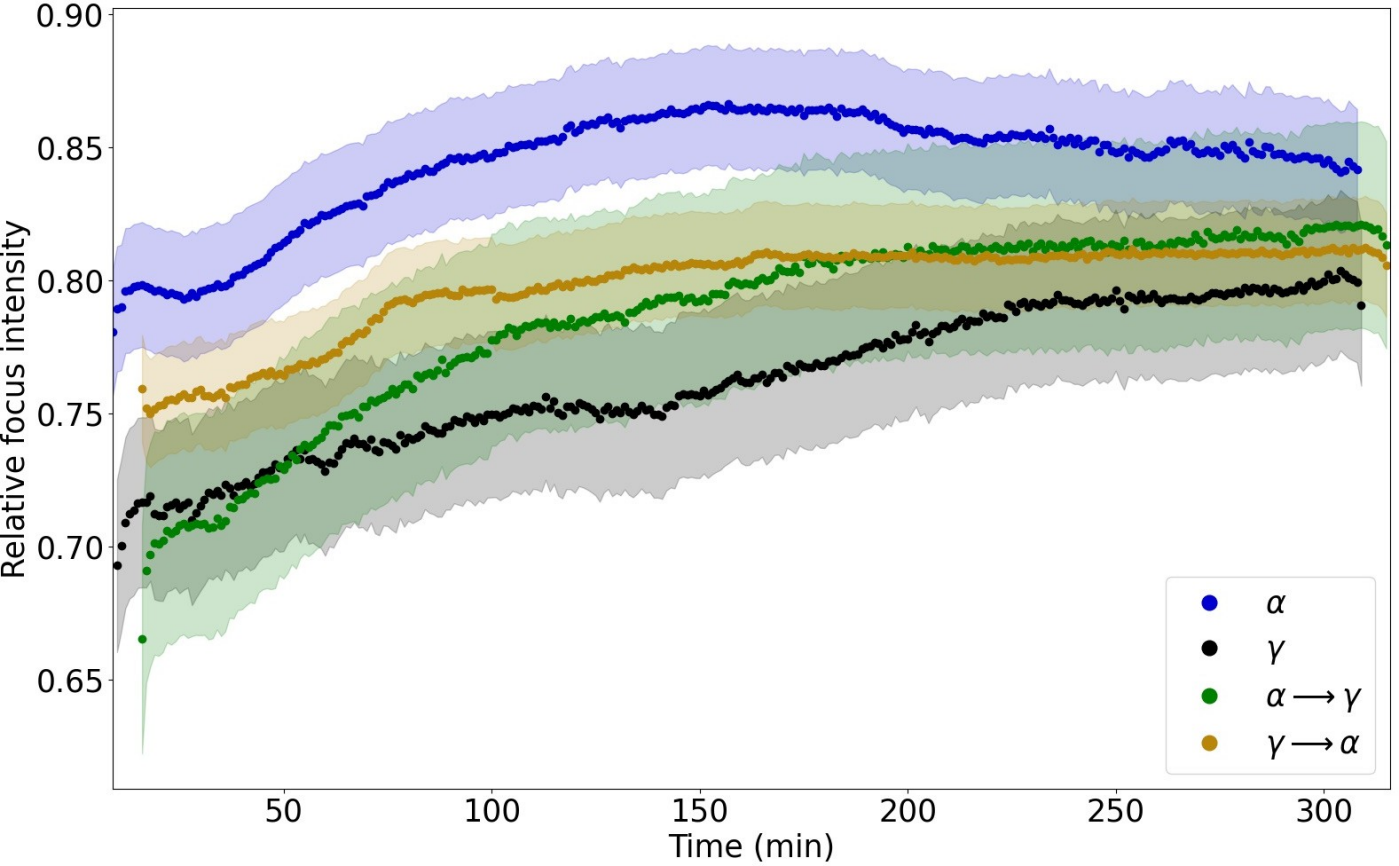
Relative focus intensities per cell nucleus during 300 min post time 0. Relative values were obtained by dividing the total focus intensities (shown in Fig 3) by the average background intensity (the mean intensity of nuclear area without foci from which the mean total focus intensity was subtracted). Corridors represent standard errors of the mean.

In order to analyse the kinetics of RIF intensities in relation to the nucleus background, we divided each mean RIF intensity by the off-focus background intensity of the nucleus (Fig 4). The highest relative RIF intensity was induced by α which can be explained by the induction of complex damage combined with a low background intensity around the RIF due to lack of sparsely ionizing hits and, consequently, the sequestration of free NBS1-GFP proteins into damaged DNA and chromatin regions resulting in less protein outside RIF [46]. γ and α → γ RIF showed similarly low relative intensities up to 60 minutes post time 0. Thereafter, the relative average γ RIF intensity remained lowest, while the intensity of α → γ RIF increased, slightly surpassing that of γ → α after ca 130 min. Overall, the relative RIF intensities of both γ → α and α → γ were intermediate to those of γ and α. For all types of radiation, the intensity of the foci gradually increased for about 120–180 min and then reached a plateau. Analysis of the dynamics of the relative RIF intensity up to 120 minutes post time 0 (based on the slopes of the linear function fits; see S3 Appendix for the slope parameters and p-values) revealed the greatest relative intensity increase for foci induced by α → γ (a = 9 ± 12 × 10^−4^), and the smallest for γ (a = 4.3 ± 8.4 × 10^−4^), a statistically significant difference.

During the first 120 min post time 0, the relative intensity value of α → γ RIF was significantly lower than of γ → α RIF (Table B1 in S2 Appendix). The difference disappeared thereafter. The slope coefficients of the fit lines to the data differed significantly for the pairs: γ : α; γ : α → γ; and γ → α : α → γ.

### Focus mobility

**Fig 5:**
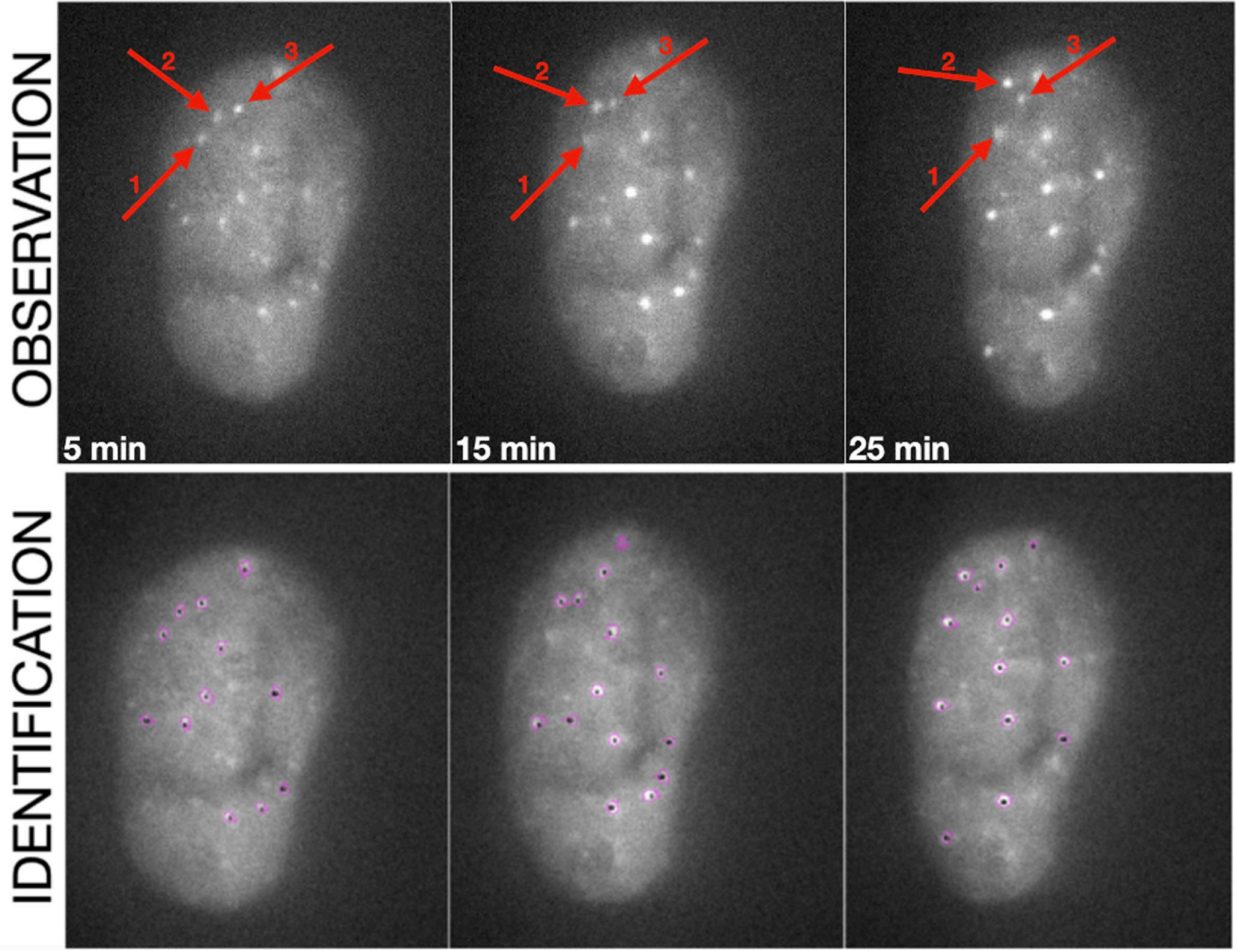
Exemplary images of nuclei recorded at given selected time points with foci induced by alpha radiation. Selected time-frames are shown. Top row: raw images of nuclei with foci. Bottom raw: same images as in the top row but with foci identified by the software. The image montage was done using the ImageJ 1.53r, version (https://imagej.nih.gov). Three repair foci and their positions in the 5^th^, 15^th^, and 25^th^ minute of observation are marked in top row images to demonstrate the upward movement of focus nr 2. Exemplary films demonstrating focus kinetics after γ → α and α → γ can be found the link: https://github.com/anna-irda/nbs1/wiki.

**Fig 6:**
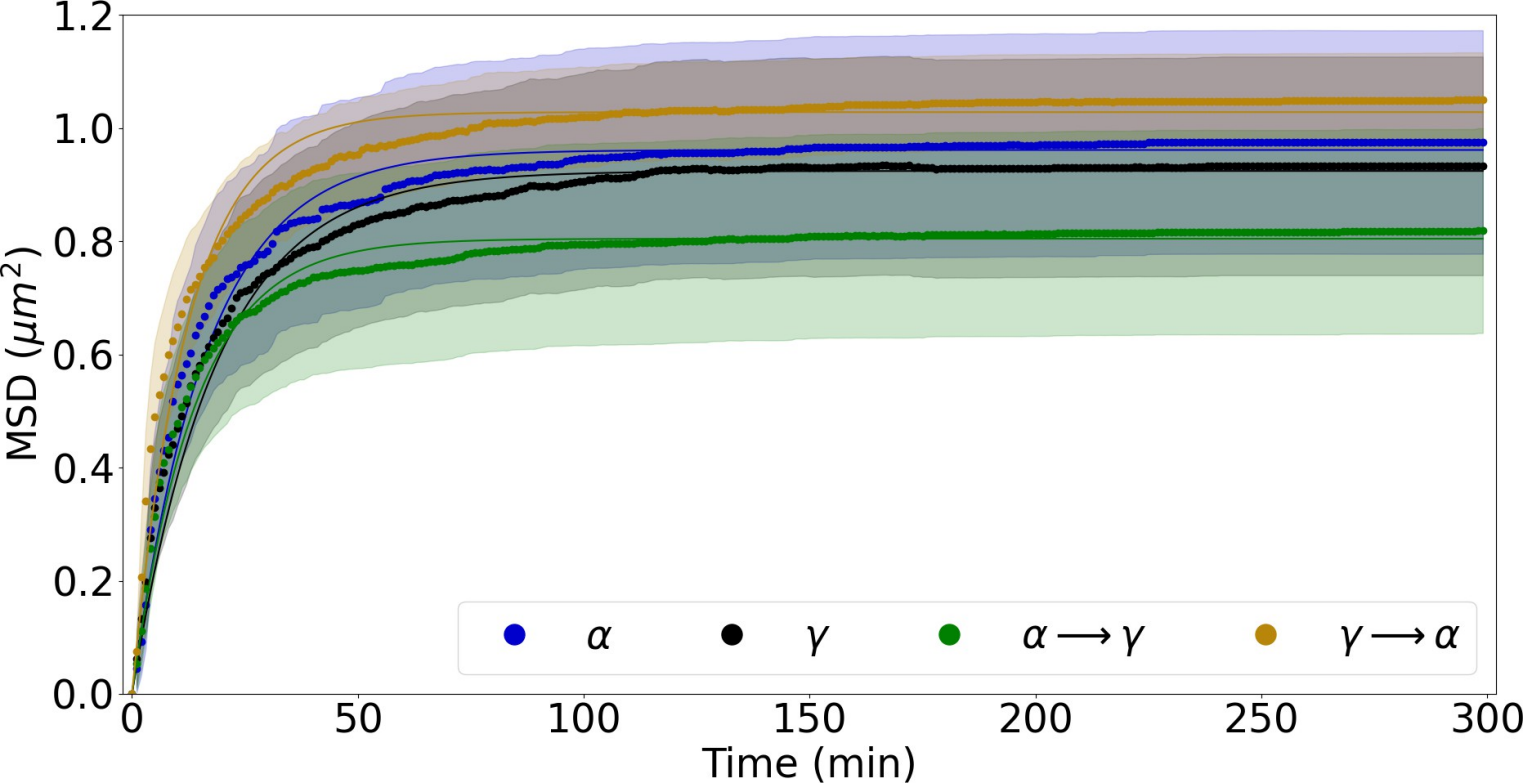
Mean square displacement (MSD) of RIF induced by alpha radiation, gamma radiation, and mixed beams of both sequences. Solid lines represent fits to data points. Corridors represent standard errors of the mean.

To investigate NBS1-GFP focus mobility over time, we tracked the motion of RIF on recorded series of images (Fig 5). The coordinates of a RIF at a given time were used to calculate their mean square displacement (MSD), which is the deviation of the position of a focus with respect to a reference position, here the centre of mass of the nucleus [32]. By determining the centre of mass of the cell nucleus for each point in time, it was possible to eliminate the impact of nucleus movement in the live cell chamber during the recording time. The results are presented in Fig 6 and the numerical data are summarized in Table 3. Two parameters of RIF mobility were determined based on the experimental data according to equation (3): the diffusion coefficient, as a measure of focus mobility and the diffusion radius as the actual radius within which this focus moved.

**Table 3:** Mean square displacement analysis with parameters for fitting the function of the sub-diffusion model. Radiation types are ranked according to the product of D_c_ and r_c_ (divided by 1000 for clarity). All parameters describing the focus movement for γ → α are significantly higher from the others (p < 0.0001 with one-tailed t-test).

Radiation type	Diffusion coefficient (D_c_ ± SEM) (nm^2^ s^-1^)	Diffusion radius (r_c_ ± SEM) (nm)	Product of D_c_ and r_c_ (divided by 1000)
γ → α	349.0 ± 6.4	1014 ± 10	354
α	242.1 ± 3.4	980.5 ± 6.7	237
α → γ	240.8 ± 3.6	897.0 ± 5.8	216
γ	206.0 ± 2.5	961.4 ± 6.0	197

RIF induced by all radiation types showed rapid, locally restricted, mobility during the first 40–50 minutes post time 0, then reaching a plateau (Fig 6). This behaviour is indicative of chromatin mobility in a sub-diffusive, locally constrained manner, with the constraint likely mediated by nuclear CCCTC-binding factor (CTCF)-flanked chromatin domains [50] containing DSBs.

The diffusion coefficients and radii are shown in Table 3 along with the product of both parameters. The product allows to rank the RIF mobility considering both parameters that are directly related to each other. RIF induced by γ → α showed the highest value, indicating that the local chromatin environment is strongly relaxed by this order of sequential mixed beam irradiation. This result is in line with the MSD results. The other radiation types were characterised by similar levels of the D_c_ and r_c_ product, being overall distinctly lower than that of γ → α .

## Discussion

The DDR triggered by ionizing radiation depends on the radiation quality. High LET radiation produces more complex damage than low LET radiation that is problematic for the cell to repair [24, 36, 51]. Also, with increasing DNA damage complexity DSB repair becomes increasingly dependent on DSB resection [4]. In this study, high LET radiation caused fewer NBS1 foci per cell as compared to low LET radiation at a similar dose, which was expected based on patterns of dense ionisations induced by alpha radiation being consistent with previously published data on gamma-H2AX foci [43] and 53BP1 foci [42] induced by mixed beam irradiation. High LET-induced foci contain DSBs at high density [24, 33, 34, 36, 52], which was reflected in our results by large focus areas and by high total as well as relative focus intensities.

In a number of earlier studies, we have shown that simultaneous exposure of cells to high and low LET radiation induces complex damage that is difficult to repair, leading to levels of damage and DDR activation that is higher than expected based on assumed additivity [39, 40, 42–44, 47]. We have speculated that the mechanism of the interaction could be based on an opening of chromatin structure by the high LET radiation-induced damage making the DNA more susceptible to attack by radicals generated by the low LET radiation or engagement of the DDR in repair of high LET-induced complex lesions to such an extent that low LET damage is not properly repaired [41]. A possible approach to test these hypotheses is to irradiate cells with low and high LET radiation not simultaneously but in alternating sequence. A DDR induced by exposure to α → γ that is stronger than following exposure to γ → α would support both hypotheses. Indeed, the obtained parameters for the dynamics of NBS1 foci in the current study showed more pronounced responses to mixed beams for α → γ IR, while the foci response to γ → α IR was generally weaker.

Both orders of sequential irradiation showed a similarly high initial number of foci, comparable to γ-irradiated cells, but for γ → α the decrease in NBS1 foci over time was more rapid than for α → γ. The initial number of foci for both mixed beam sequences was higher than the expected level calculated on assuming additivity between γ and α. This observation is in accordance with earlier results for gamma-H2AX [43] and 53BP1 [42] in cells exposed to high and low LET radiation simultaneously and can be interpreted as resulting from synergistic action of both radiations possibly due to a high sensing of DNA damage induced by the mixed beam. It appears unlikely that α → γ makes the DNA more susceptible to attacks by radicals generated by the low LET radiation because in such a case the level of initial foci would be higher in α → γ than in γ → α. The lifetime of radicals in water is shorter than a second [53] so synergism at the level of DNA damage induction due to this mechanism should be reflected by foci formed already during exposure and very shortly thereafter. Interestingly, in earlier studies with simultaneous exposure to low and high LET radiation, a similar level of initial mixed beam-, and low LET-induced 53BP1 foci was only observed when analysis was carried out in fixed cells [42], but not by live microscopy [47]. We attributed this difference to a higher lateral microscopic resolution due to flattening of the nuclei during fixation and detergent extraction associated with immunofluorescence procedures for fixed cell analysis. It is also possible that the NBS1 foci, being smaller than 53BP1 (own observation), are a more sensitive parameter of the DDR in a 2D setup. Comet assay using peripheral blood lymphocytes also confirmed synergy in the accumulation of initial DNA breaks (net relative comet tail intensity) for mixed beam exposure [39] and so did the analysis of gamma-H2AX foci in fixed human fibroblasts [43].

The synergy between γ and α in terms of delayed repair was only seen for α → γ. For γ → α there was rapid repair with focus numbers falling below the expected line and nearly reaching the number of foci induced by α. This indicates that following γ → α the simple DSBs induced by γ were rapidly repaired leaving only the complex damage at the end of the observation interval of 300 min. The fast decay of foci per nucleus during 50–100 min of repair suggests that the presence of low LET-induced damage before that of high LET speeds up the DDR. This reaction is fascinating, but the mechanism is not clear. It could relate to the priming of the apical kinases of the DDR by a low LET pre-dose, or to a loosened chromatin structure at 5 min after sparsely ionizing γ, which could allow a faster repair of the α-induced damage, similarly as seen after histone deacetylase inhibition [29]. Following α → γ, the repair was slower, even than that of γ alone, and this delayed repair pattern was more similar to results from our previous studies using simultaneous mixed beams exposures [37, 38, 40, 42–44, 47]. In these experiments, radiation exposure with alpha particles and X-rays started at the same time, but due to a higher dose rate of alpha radiation compared to X-rays, X-ray exposure lasted longer. Due to the higher dose rate of the gamma source used in the present study as compared to the X-ray source used in the previous studies (around 0.4 versus 0.05 Gy/min, respectively), the total time for sequential or combined exposure was similar in both studies, or even shorter in the current sequential setting. Therefore, it does not seem to matter whether the high LET component is present before or simultaneously with the low LET counterpart: the outcome is an interaction of both radiations that is expressed as initial damage (at least within the observation period that started few minutes after termination of exposure) and delayed repair. The results support the assumption that high LET-induced complex lesions engage the DDR to such an extent that low LET damage is not repaired efficiently, or at later time points beyond the time frame examined in this study.

To further address mechanistic aspects of the cellular response to mixed beams, we analysed focus areas, where complex damage manifests itself as large foci [34, 54, 55], as well as the total focus intensities, which may be indicative of the total load of NBS1 protein at a particular DSB site [36]. In both cases, there was a significant increase after 120 min of observation in the case of α → γ. An increase of focus areas may indicate the accumulation of damaged DNA segments that are difficult to repair and attract higher numbers of DDR proteins or that are congregated to facilitate repair, with merging of foci having been observed during the repair process [56]. It may also relate to remaining foci undergoing slower repair in processing of complex damage and/or being located in repair-inhibiting heterochromatin. High LET radiation can induce a more or less prominent biphasic response in different cell systems, where the second peak in focus number at around 3–6 h post exposure corresponds to the processing of heterochromatic damage, related to ATM-KAP1-dependent repair [57]. In U2OS cells, we did not see a biphasic response for NBS1 foci number in the time frame analysed, which is consistent with previous studies of 53BP1 where we only saw a minor elevation at 4–5 h [42]. However, for focus area a small flattening curve was visible for alpha radiation at 200 min, and a large peak was present for α → γ at around 240 min (corresponding to 4 h).

When the relative focus intensity was calculated, α was highest and γ lowest, which is in line with previous work in which high LET radiation was reported to induce a high 53BP1 focus intensity compared to low LET damage, interpreted as a high local concentration of repair proteins as well as the presence of many individual resection events, determined by multiple replication protein A (RPA) foci per 53BP1 chromatin focus [36]. Resection-dependent repair is preferentially activated at site of on complex DSBs [4]. Since our relative intensity measurements were based on subtracted background levels, it is not surprising that α, which only has a minor sparsely ionizing component, is higher than mixed beams in relation to its background levels. It may also be relevant to mention that high LET induced damage may sequester a large amount of some DDR components [35] thereby influencing repair outcomes.

Our MSD measurements revealed that a plateau pattern was reached after the rapid initial mobility of foci for all types of radiation, indicating subdiffusive motion of the DSB carrying NBS1-tagged foci and subnuclear motility in the confined space [32]. The data also agree with results of Jakob et al. who noted a limited LET-independent mobility of DSB repair foci in the chromatin of exposed cells [58].

The similar diffusion coefficients observed for α and α → γ suggest that an α-hit chromatin environment dominates the DDR with respect to NBS1 focus mobility even when scattered gamma-induced DSBs are added. Among the radiation schemes tested, γ → α displayed the highest MSD and the highest diffusion radius.

The low level of focus movement (MSD) for α → γ as compared to γ → α suggests an involvement of chromatin in this response. The opening of chromatin at many places throughout the nucleus by low LET radiation, just before high LET exposure, as well as the rapid increase in movement during the first 40–50 min are correlated in time with the fast repair of a part of the complex damage induced by high LET radiation. This may be due to the low LET-induced formation of simple DSBs largely in a euchromatic environments whose repair is driven by NHEJ and not restrained by compacted heterochromatin [24, 59]. This is supported by our earlier results in breast cancer cells, where we demonstrated that chromatin opening by histone deacetylase inhibitor induced a faster repair of γH2AX foci after alpha radiation exposure, along with a net radioprotective effect on cell survival [29]. Earlier studies show that for complex damage occurring in heterochromatin, DSBs must move toward the heterochromatin/euchromatin border to be properly repaired [60]. It can be considered that high LET radiation leads to a different distribution of damage according to chromatin environment, in particular in some nuclear regions, since tracks traversing the nucleus will hit both eu- and heterochromatin and since DSBs induced in euchromatin are more rapidly repaired than when induced in heterochromatin [28, 57, 60]. It is also possible that in the case of high LET radiation first, the complex DSBs will quickly recruit repair factors, while the subsequently induced low LET-induced simple damage may soak up the remaining free pool of limited factors which may change the repair pathway choice [35, 36]. In this context it is interesting to mention the observation of [30] that chromatin mobility is strongest at sites of highly complex DNA damage. The latter may relate to the dissolution of chromatin architecture along high LET particle tracks as seen by TEM analysis [61].

Since our present analysis was restricted to analysis of NBS1 foci and it is not possible to predict the impact of the observed differences in focus kinetics on radiation-induced cell death. However, a number of earlier clonogenic cell survival studies, in various cell types sequentially exposed to high and low LET radiation in alternating order, revealed a synergistic effect between both radiation qualities only when the high LET exposure preceded the low LET exposure [26, 62–67]. In contrast, only two studies claimed that the order of exposure has no influence on the occurrence of synergism [68, 69]. Thus, the majority of the clonogenic cell survival data are supportive of our results.

Finally, and since our current study was focussed on the 5 h time frame after irradiation it would be interesting to follow the focus behaviour for more extended time periods. In our setup this was not possible due to bleaching of the fluorescence signal and detachment of cells at the end of the observation period, possibly caused by eventual phototoxic effects by the frame rate used and culture conditions in the live imaging chamber. An additional interesting approach would be to follow the formation of foci in 3D by confocal microscopy. Due to the orthogonal configuration of the α exposure setup, one α-induced focus represents one particle traversal that could possibly induce several foci along its track, as was shown by [70]. 3D analysis would allow analysing foci in the Z axis which we were not able to detect due to their overlapping.

The study has several limitations. Due to the limited resolution of the applied system, it was not possible to show DNA damage in the context of chromatin conformation. This limitation could be overcome by applying higher resolution, such as i-BLESS [71]. In addition, cells were not synchronized with respect to the cell cycle. Hence, the presented results cannot be related to the pathway of DSB repair [20–22]. This limitation could be overcome by synchronizing cells prior to radiation exposure.

## Conclusions

In conclusion, our study demonstrates that in the case of sequential exposure to mixed beams of high and low LET, the order of irradiation differentially influences the DDR. It appears that following exposure of cells to sequential high and low LET radiation, the DDR engages in the repair of high LET radiation-induced complex lesions to such an extent that low LET radiation-induced damage is repaired with a delay. This result provides insight into the spatiotemporal organisation of DNA repair processes which seems to be modulated by the presence of damage of different complexity within a cell nucleus.

## Supporting information

S1 Appendix focus frequency studies. (DOCX)

S2 Appendix focus area studies. (DOCX)

S3 Appendix focus intensity studies. (DOCX)
