# Supplementary material for: The order of sequential exposure of U2OS cells to gamma and alpha radiation influences the formation and decay dynamics of NBS1 foci
Source: PLoS One. 2023 Jun 12;18(6):e0286902. doi: 10.1371/journal.pone.0286902 (PMC10259794; doi:10.1371/journal.pone.0286902)
Supplement: S1 Appendix — (DOCX) [file pone.0286902.s001.docx]

**S1 Appendix: focus frequency studies**

Fig A1. NBS1 focus frequency in cells exposed to mixed beams of alpha and gamma radiation for the first 90 minutes with the fitted linear function. The slope parameters a are given in S1 Table.

Table A1. Linear function parameters with their uncertainties fitted to focus frequency for both mixed beam irradiation scenarios analysed for the first 90 minutes (no statistically significant difference).

| **Radiation type** | **Slope parameter (𝒂) with its uncertainty (1/min)** |
| --- | --- |
| 𝛼→𝛾 | -0.13 ± 0.31 |
| 𝛾 → 𝛼 | -0.23 ± 0.41 |
| **p-value** | 0.0666 |
