# Supplementary material for: The order of sequential exposure of U2OS cells to gamma and alpha radiation influences the formation and decay dynamics of NBS1 foci
Source: PLoS One. 2023 Jun 12;18(6):e0286902. doi: 10.1371/journal.pone.0286902 (PMC10259794; doi:10.1371/journal.pone.0286902)
Supplement: S2 Appendix — (DOCX) [file pone.0286902.s002.docx]

**S2 Appendix: focus area studies**


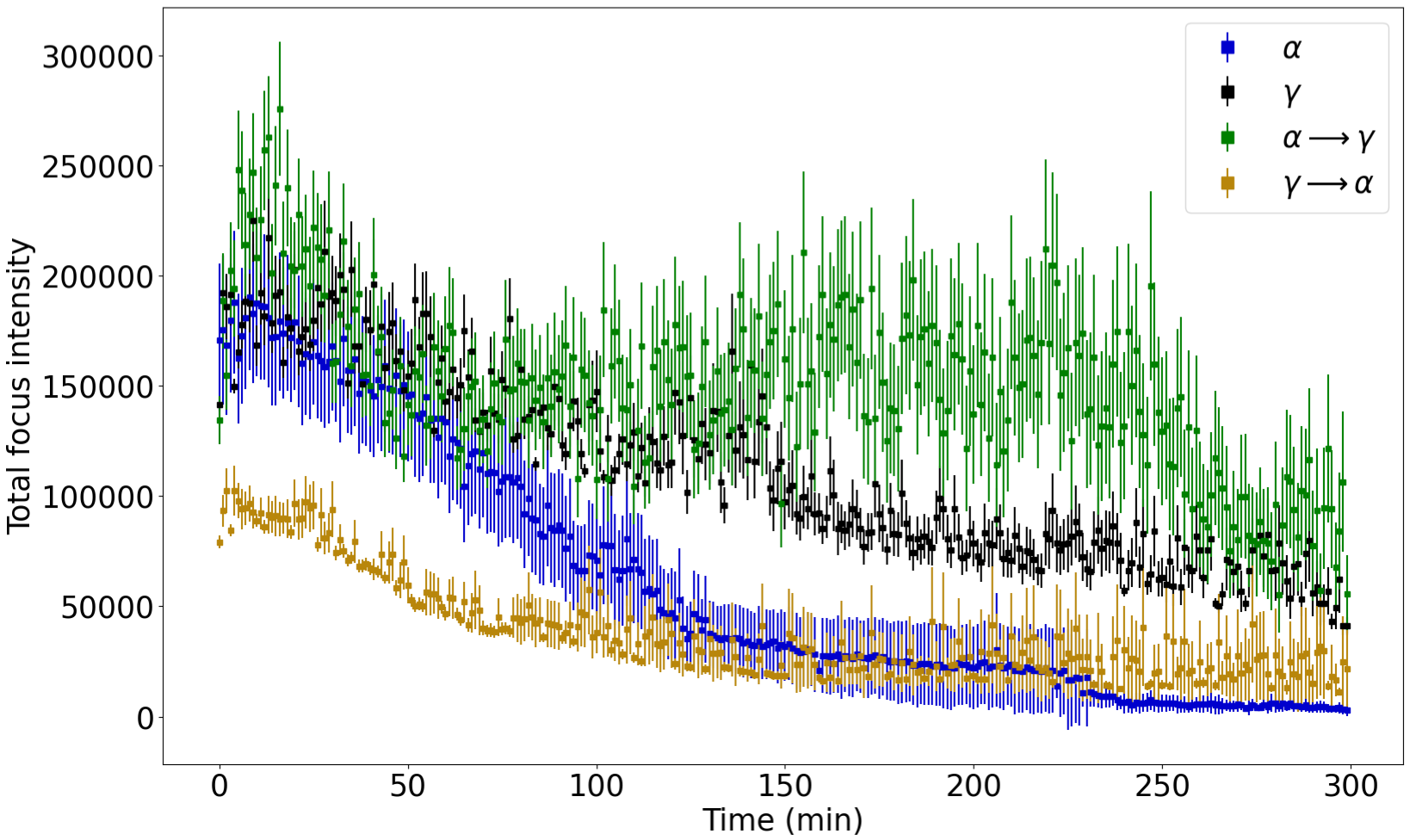


Fig B1. The mean area of foci induced in irradiated cells as a function of time. Error bars represent standard errors of the mean (SEM).

Table B1. Parameter values of polynomials fitted to focus area as the function of time.

| **Radiation type** | $\boldsymbol{a}_{\boldsymbol{0}}$ | $\boldsymbol{a}_{\boldsymbol{1}}$ | $\boldsymbol{a}_{\boldsymbol{2}}$ **(10^-5^)** | $\boldsymbol{a}_{\boldsymbol{3}}$  **(10^-6^)** | $\boldsymbol{a}_{\boldsymbol{4}}$ **(10^-8^)** | $\boldsymbol{a}_{\boldsymbol{5}}$  **(10^-10^)** | $\boldsymbol{a}_{\boldsymbol{6}}$  **(10^-12^)** | $\boldsymbol{a}_{\boldsymbol{7}}$ **(10^-15^)** | $\boldsymbol{a}_{\boldsymbol{8}}$ **(10^-18^)** |
| --- | --- | --- | --- | --- | --- | --- | --- | --- | --- |
| 𝛼 | 1.254 | -0.00426 | 5.46 | 2.5 | 8.162 | 8.449 | -4.078 | 9.421 | -8.42 |
| 𝛾 | 0.2507 | 0.039 | -168.6 | 32.1 | -32.9 | 19.46 | -6.671 | 12.31 | -9.46 |
| 𝛼→𝛾 | -0.2449 | 0.09483 | -314.5 | 48.59 | -39.56 | 17.79 | -4.245 | 4.53 | -1.065 |
| 𝛾 → 𝛼 | -0.7916 | 0.1271 | -464.1 | 83.49 | -83.16 | 48.03 | -15.98 | 28.41 | -20.88 |
