# Supplementary material for: The order of sequential exposure of U2OS cells to gamma and alpha radiation influences the formation and decay dynamics of NBS1 foci
Source: PLoS One. 2023 Jun 12;18(6):e0286902. doi: 10.1371/journal.pone.0286902 (PMC10259794; doi:10.1371/journal.pone.0286902)
Supplement: S3 Appendix — (DOCX) [file pone.0286902.s003.docx]

**S3 Appendix: focus intensity studies**


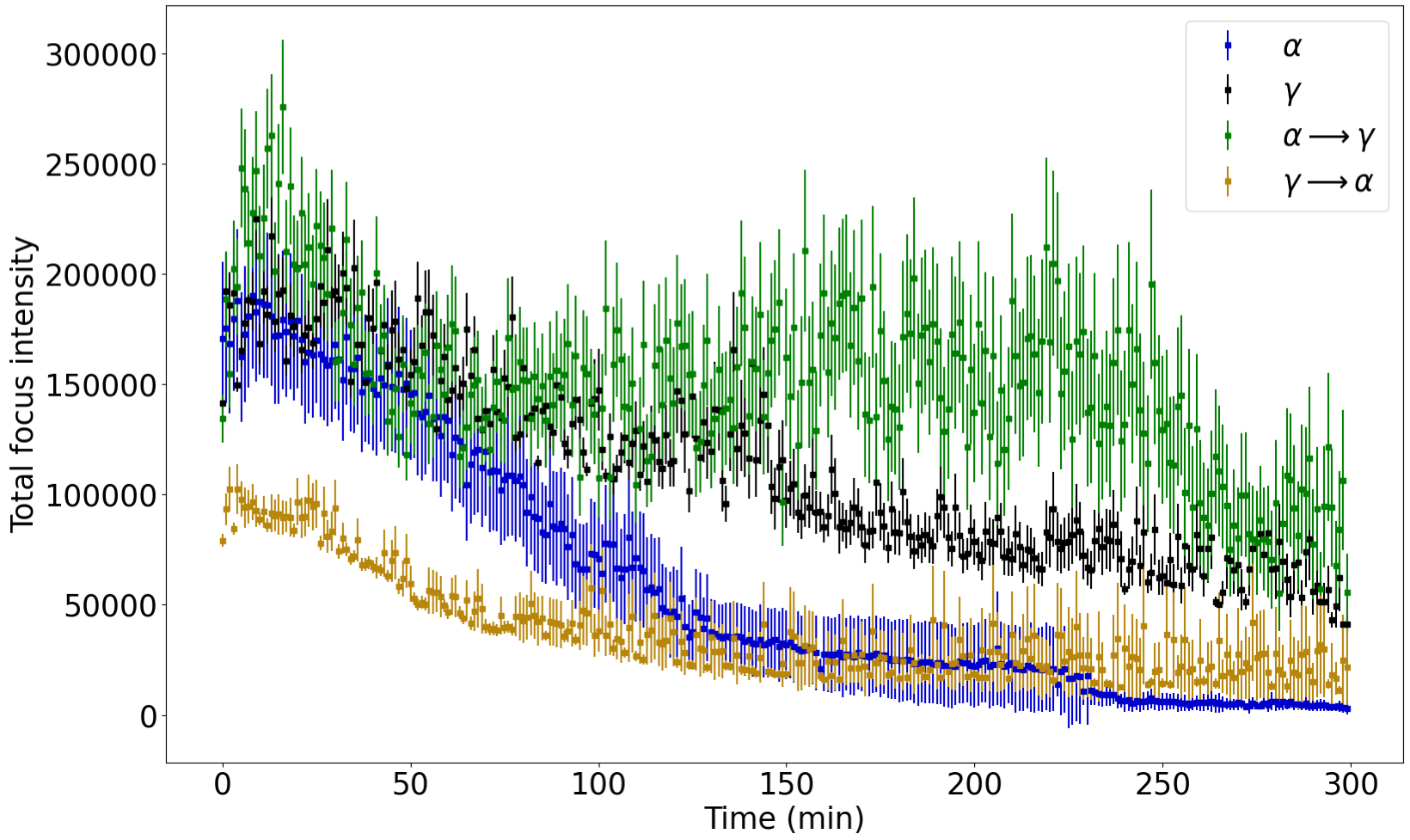


Fig C1. The sum of the intensities of pixels located in the repair foci per cell nucleus.

Table C1. Fit parameters of polynomial functions.

| **Radiation type** | $\boldsymbol{a}_{\boldsymbol{0}}$ **(104)** | $\boldsymbol{a}_{\boldsymbol{1}}$ | $\boldsymbol{a}_{\boldsymbol{2}}$ | $\boldsymbol{a}_{\boldsymbol{3}}$ | $\boldsymbol{a}_{\boldsymbol{4}}$  **(10-3)** | $\boldsymbol{a}_{\boldsymbol{5}}$  **(10-6)** | $\boldsymbol{a}_{\boldsymbol{6}}$**(10-9)** |
| --- | --- | --- | --- | --- | --- | --- | --- |
| 𝛼 | 13.98 | 3585 | -104.1 | 0.97 | -4.3 | 9.4 | -8.1 |
| 𝛾 | 10.93 | 5074 | -133.3 | 1.40 | -7.3 | 18.4 | -18.12 |
| 𝛼→𝛾 | 11.69 | 6739 | -187.7 | 2 | -10.0 | 23.4 | -21.27 |
| 𝛾 → 𝛼 | 7.504 | 1541 | -54.29 | 0.57 | -2.8 | 6.7 | -6.213 |


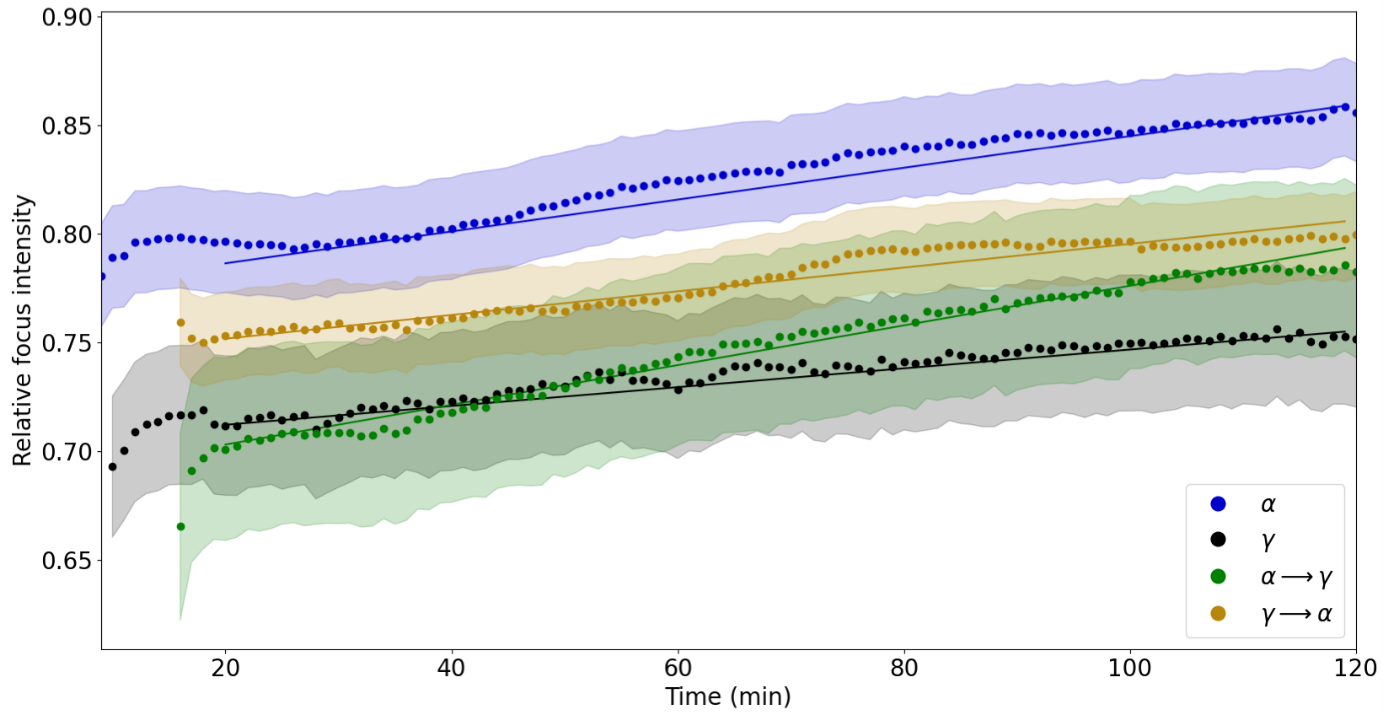


Fig C2. The relative intensity with the fitted linear functions to the first 120 minutes.

Table C2. The slope of the line from fitting the linear function to the first 120 minutes of the intensity-time course.

| **Radiation type** | **Slope parameter with its uncertainty** 𝒂 ± 𝒖𝒂 **(10-4)** |
| --- | --- |
| 𝛼 | 7 ± 10 |
| 𝛾 | 4.3 ± 8.4 |
| 𝛼→𝛾 | 9 ± 12 |
| 𝛾 → 𝛼 | 5 ± 15 |

Table C3. Significance values (p-values) in difference between the observed means in two samples of irradiated cells. *significantly different

| **Radiation type** | **𝛼** | **𝛾** | **𝛼 → 𝛾** | **𝛾 → 𝛼** |
| --- | --- | --- | --- | --- |
| **𝛼** |  | 0.0244* | 0.1620 | 0.2255 |
| **𝛾** | 0.0244* |  | 0.0005* | 0.6560 |
| **𝛼 → 𝛾** | 0.1620 | 0.0005* |  | 0.0234* |
| **𝛾 → 𝛼** | 0.2255 | 0.6560 | 0.0234* |  |
